# Blood transcriptional profiling reveals IL-1 and integrin signaling pathways associated with clinical response to extracorporeal photopheresis in patients with leukemic cutaneous T-cell lymphoma

**DOI:** 10.18632/oncotarget.26900

**Published:** 2019-05-07

**Authors:** Zuolin Ying, Lisa Shiue, Katherine Park, Jutta Kollet, Pedram Bijani, Meghali Goswami, Madeleine Duvic, Xiao Ni

**Affiliations:** ^1^Department of Dermatology, The University of Texas MD Anderson Cancer Center, Houston, TX 77030, USA; ^2^Bioinformatics, Miltenyi Biotec GmbH, Beigisch Gladbach, 51429, Germany

**Keywords:** non-Hodgkin’s lymphoma, cutaneous T-cell lymphoma, extracorporeal photopheresis, microarray, integrin

## Abstract

Extracorporeal photopheresis (ECP) is a frontline therapy for patients with leukemic cutaneous T-cell lymphoma (L-CTCL), but its mechanisms of action are not fully understood. This study was to explore the molecular mechanisms underlying clinical response versus non-response in patients with L-CTCL. We performed blood transcriptional profiling of ten L-CTCL patients at Day 2 and 1 month post- ECP compared to pre-ECP baseline using Agilent Whole Human Genome Microarray technology. Differentially expressed genes (DEGs) between five clinically-responsive patients and five clinically-resistant patients were cross-compared. Higher numbers of genes were modulated in responders than non-responders after ECP at both Day 2 and 1 month, with two thirds of DEGs down-regulated. The down-regulated DEGs at 1 month post-ECP were related to inflammatory, immune and/or stress responses, platelet functions, and chromatin remodeling. Upregulated DEGs were mainly related to functions of the nucleolus. Pathway analysis revealed that integrin and IL-1 signaling pathways were the top pathways affected in responders, which were minimally affected in non-responders. The top upstream transcription regulators affected were IL1B, EGR1, FAS, and TGFB1. Our results suggest that the modulation of cell adhesion and suppression of IL-1β induced inflammation may underlie the efficacy of ECP in L-CTCL.

## INTRODUCTION

Cutaneous T-cell lymphomas (CTCL) are a group of lymphoproliferative diseases characterized by clonal skin-homing malignant helper T cells [1]. Mycosis Fungoides (MF) and Sézary Syndrome (SS) are the most common variants of CTCL. SS with diffuse erythroderma and MF harboring clonal malignant T cells in the blood are considered as leukemic CTCL (L-CTCL) [2]. Clonal malignant T cells in MF/SS possess a mature memory T-cell phenotype and are mostly CD4^+^CD26^–^ and/or CD4^+^CD7^–^ [3]. Although the pathogenesis of CTCL remains unclear, defective apoptosis, chronic inflammation, and immunosuppression are thought to be involved. A defective signaling in FAS/FAS ligand pathway is an early cause of malignant T cells failing to undergo activation-induced cell death (AICD) [4]. Skin inflammation and epidermotropism of malignant T cells are attributed to the over-expression of cytokines (IL-1β, IL4), cutaneous lymphocyte-associated antigen (CLA), skin-homing chemokines (CCR4, CCR10), and adhesion molecules [5–9]. Adhesion molecules not only mediate cell attachment, but also initiate signaling and participate in the formation of “immunological synapses” between T cells and antigen presenting cells (APCs) [10]. Pautrier’s microabscesses, seen in MF, consist of Langerhans cells in contact with epidermal T cells. Multiple integrins, as key adhesion molecules, are involved in regulating T-cell migration and function [11].

Extracorporeal photopheresis (ECP) is an effective frontline therapy for patients with L-CTCL [12]. ECP is an apheresis procedure in which leukocytes are exposed *ex vivo* to 8-methoxypsoralen (8-MOP) and UVA radiation, and then reinfused to patients. The overall response rate of ECP in CTCL patients is between 54% and 74% with a 14%–33.3% complete response rate [13–15]. Mechanisms of action of ECP in CTCL includes induction of apoptosis in malignant T cells, promotion of monocytes to dendritic cell differentiation, reversal of cytokine imbalance, and immunomodulatory effects [16, 17]. Our group has previously reported that increases in myeloid and plasmacytoid dendritic cells in the blood occurred after ECP therapy in patients with L-CTCL [18]. We also found that SS patients with CD4^+^CD25^–^Foxp3^+^ malignant T cells are more likely to respond to ECP therapy [19]. It was recently reported that αVβ3 and α5β1 integrin signaling may participate in driving monocyte to dendritic cell conversion in two model systems of ECP [20]. Nevertheless, molecular signaling pathways in patients with L-CTCL following ECP remain largely unknown, which prevents the tailoring of ECP for more effective clinical use.

The purpose of this pilot study was to explore the molecular mechanisms underlying the efficacy of ECP in L-CTCL in more detail. We used Agilent Whole Human Genome Microarrays to assess transcriptional profiles in peripheral mononuclear blood cells (PBMCs) of ten L-CTCL patients at Day 2 and 1 month post-ECP compared to baseline. Differentially expressed genes in five clinically-responsive patients (responders) were compared to five clinically-resistant patients (non-responders). Canonical biological pathways were analyzed using Ingenuity Pathway Analysis. Many differentially expressed genes, transcription regulators, and biological pathways in clinically-responsive patients were identified that distinguished them from clinically resistant L-CTCL patients.

## RESULTS

### Patient demographics

Table 1 shows the demographics of ten patients in this study, who were part of our previously reported studies [2, 18]. Eight of 10 (80%) patients were Caucasians, 9 of 10 (90%) were at stage IV, and the median age was 66.5 (54–78) years. The median number of ECP administered was 8.5 (6–13) in a 6-month course of treatment. Patients received 1 or 2 cycles of ECP at 1 month. All patients initially received ECP monotherapy, and 8 of 10 patients were in combinational therapy at 3 months later. Following a 6-month course of treatment, clinically-responsive patients (responders, R, *n* = 5) had a dramatic skin improvement with an average of 60.9% decrease in mSWAT scores while clinical-resistant patients (non-responders, NR, *n* = 5) had a 17% increase in mSWAT scores. There were no significant differences between responders and non-responders in age, sex, tumor burden, and skin involvement before ECP therapy.

**Table 1:** Demographics of L-CTCL patients

	Pt.#	Age/Sex/Race	Stage	TCRvβ (%)	mSWAT	Sézary cells		ECP cycles		Additional therapy (at 3 months)	Response
						%	/μl	at 1 month	at 6 months		
**Non- responders (*n* = 5)**	1	74/M/C	MF IVB	Vβ1 (98.0)	95.5	57.2	627	1	7	Bexarotene, IFNα	PD
	2	71/M/AA	SS IVB (HTLV+)	None	100.0	55.5	525	1	7	Bexarotene, IFNα	PD
	3	54/M/C	MF IVA	Vβ17 (54.0)	39.0	20.6	69	1	7	None	SD
	4	66/F/C	SS IVB	Vβ2 (97.0)	100.0	93.8	17981	2	13	Bexarotene, IFNα	SD
	5	78/F/AA	SS IVB	Vβ13.6 (95.0)	47.0	94.5	9977	2	10	None	PD
**Responders (*n* = 5)**	6	58/F/C	SS IVA	Vβ (70.0)	63.0	64.2	1751	1	7	Bexarotene, IFNα	MR
	7	66/M/C	SS/MF IIIB	Vβ22 (91.0)	100.0	82.2	413	2	10	Bexarotene, IFNα	PR
	8	74/F/C	SS IVB	Vβ (85.0)	93.0	89.9	3928	2	11	Bexarotene	PR
	9	63/M/C	SS IVB (BM+)	Vβ (94.0)	49.0	94.7	22751	2	10	IFNα	PR
	10	67/F/C	SS IVA	n/d	67.0	91.9	4300	1	6	Bexarotene	PR

Abbreviations: F: female; M: male; C: Caucasian; AA: African American; SS: Sézary syndrome; MF: mycosis fungoides; mSWAT: modified severity-weighted assessment tool; MR: minor response; PR: partial response; PD: progressive disease; SD: stable disease; n/d: not done.

### T-cell subsets and dendritic cell subsets

**Figure 1:**
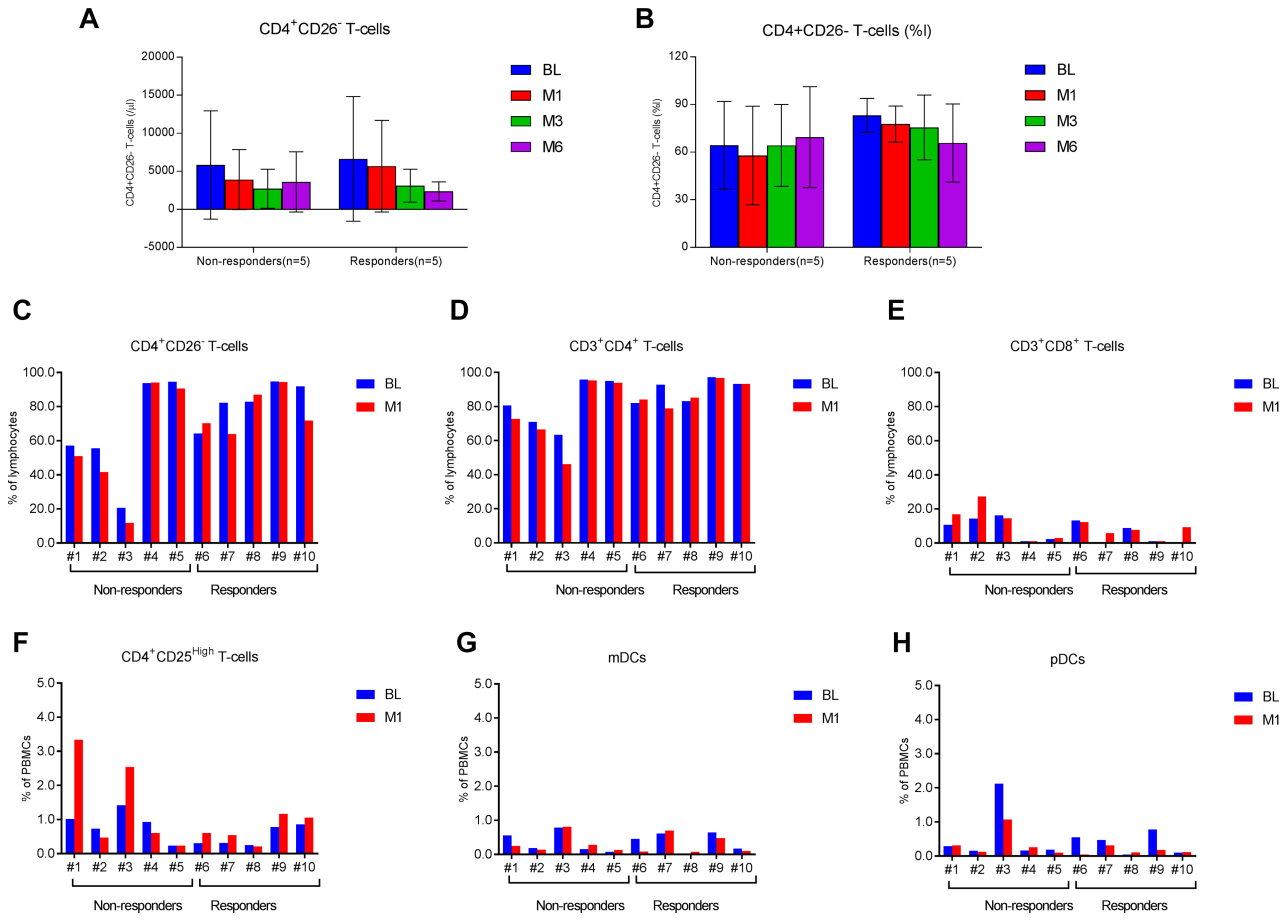
T-cell and dendritic cell subsets in L-CTCL patients before and after ECP therapy. Multi-color flow cytometry analysis was done to measure frequencies of four T-cell subsets and two dendritic cell subsets in the blood of L-CTCL patients before and after ECP therapy. The absolute counts (/μl, **A**) and percentages (% of lymphocytes, **B**) of CD4^+^CD26^–^ malignant T cells at baseline (BL), 1 month (M1), 3 months (M3), and 6 months (M6) post-ECP are presented in responders (*n* = 5) and non-responders (*n* = 5). Data are represented as mean ± SD. The percentages of CD4^+^CD26^–^ malignant T cells (**C**), CD3^+^CD4^+^ T cells (**D**), CD3^+^CD8^+^ T cells (**E**), CD4^+^CD25^high^ T cells (**F**), Lin^−^HLA-DR^+^CD11c^+^ myeloid dendritic cells (mDCs, **G**), and Lin^−^HLA-DR^+^CD123^+^ plasmacytoid dendritic cells (pDCs, **H**) at baseline and 1 month after ECP are presented for each of 10 patients in this study.

After 6 months of treatment, patients showed various changes in their peripheral blood T-cell and dendritic cell subsets. Overall, absolute counts of CD4^+^CD26^–^ malignant T cells were reduced by about 64% in responders (*n* = 5) at 6 months post-therapy in comparison to a reduction of about 38% in non-responders (*n* = 5) (Figure 1A). The reduction in percentages of malignant cells was seen only in responders following a 6-month course of ECP therapy (Figure 1B). Of note, both absolute counts and percentages of malignant T cells were decreased at 1 month (M1) of ECP therapy in most of responders and non-responders (Figure 1A–1C). After the first month, responders showed a continuous decrease of percentages of malignant T cells at 3 months and 6 months after therapy, whereas non-responders showed a reverse in percentages of malignant T cells at 3 months and 6 months post-therapy. Figure 1C–1H show the paired cell numbers (%) in PBMCs at BL and M1 for each patient in four T-cell subsets (CD4^+^CD26^–^ T cells, CD3^+^CD4^+^ T cells, CD3^+^CD8^+^ T cells, and CD4^+^CD25^High^ T cells) and two dendritic cell subsets (Lin^−^HLA-DR^+^CD11c^+^ mDCs, and Lin^−^HLA-DR^+^CD123^+^ pDCs).

### Transcriptional profiling

**Figure 2:**
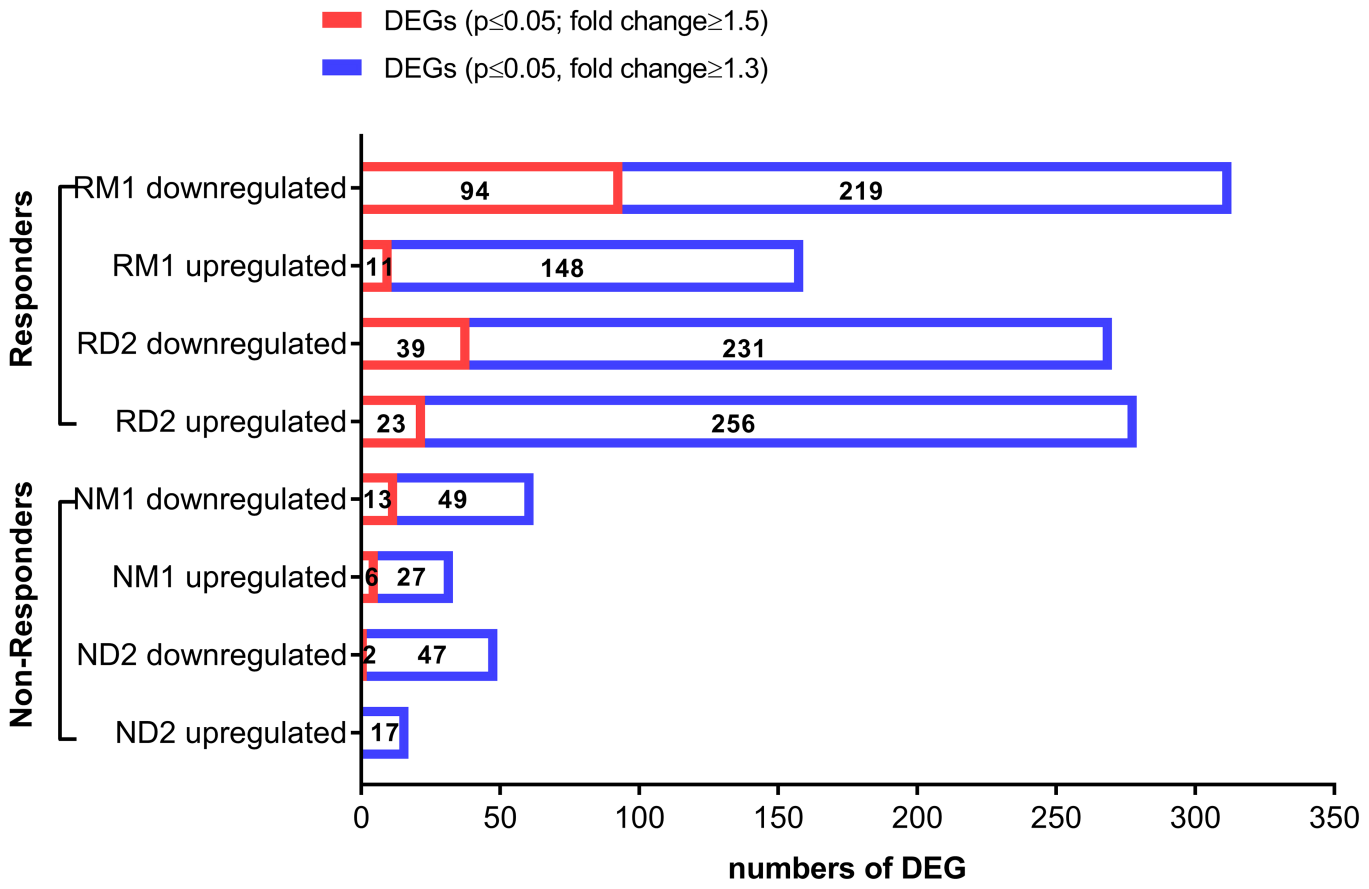
Differentially expressed genes (DEG) in clinically-responsive patients and clinically-resistant patients. Transcriptional changes were profiled using total RNA extracted from peripheral blood mononuclear cells (PBMCs) of L-CTCL patients, using Agilent Whole Human Genome Microarrays. Bioinformatic analysis of microarray data was done to identify differentially expressed genes (DEGs) for clinically-responsive patients (responders) at Day 2 (RD2) and 1 month (RM1) post-ECP and clinically-resistant patients (non-responders) at Day 2 (ND2) and 1 month post-ECP (NM1). Down-regulated and upregulated DEGs are provided for each group. The red bars indicate DEGs with *p* ≤ 0.05 and fold change ≥1.5. The blue bars indicate DEGs with *p* ≤ 0.05 and fold change ≥1.3.

To investigate transcriptional changes occurring in PBMCs following ECP therapy, we performed whole transcriptome profiling using Agilent Whole Human Genome Oligo Microarrays for Day 2 (D2), and 1 month (M1) post-ECP compared to baseline (BL) immediately before ECP. After raw ratio data pre-processing and transformation, non-logarithmic fold changes were calculated. Transcripts were considered to be differentially expressed when they had a *p*-value ≤ 0.05 plus a ≥ 1.5-fold average expression difference compared to baseline (BL). With this cutoff, a total of 165 differentially expressed genes (DEGs) were identified from four groups (RD2, RM1, ND2, and NM1). All 165 DEGs are provided in Supplementary Tables 2–8 for each group. Table 2 lists 23 DEGs which are present in two groups, with 19 DEGs in two responder groups (RD2 and RM1) while 2 DEGs were observed in RD2 and NM1 groups, and 2 DEGs in RM1 and NM1. As shown in Figure 2, overall, more genes were differentially expressed in responders (148) than in non-responders (21); furthermore, more genes were differentially expressed at 1 month (122) than at Day 2 after ECP (64). DEGs were predominantly down-regulated in both responders (116) and non-responders (15) than those up-regulated (32 in responders and 6 in non-responders). The highest numbers of DEGs (105) were seen in responders at RM1group with 94 genes down-regulated and 11 genes up-regulated.

**Table 2:** DEGs observed in 2 groups

Agilent_ID	Gene symbol	Systematic name	ND2 upregulated	ND2 downregulated	NM1 upregulated	NM1 downregulated	RD2 upregulated	RD2 downregulated	RM1 upregulated	RM1 downregulated	Summary
A_23_P111701	*GNG11*	NM_004126						1		1	
A_23_P116264	*NRGN*	NM_006176						1		1	
A_23_P122443	*HIST1H1C*	NM_005319						1		1	
A_23_P38519^*^	*ITGB3*	NM_000212						1		1	
A_23_P416581	*GNAZ*	NM_002073						1		1	
A_23_P501831	*C5orf4*	NM_032385						1		1	
A_23_P51136	*RHOB*	NM_004040						1		1	
A_23_P77971^**^	*ITGA2B*	NM_000419						1		1	
A_23_P93258	*HIST1H3B*	NM_003537						1		1	
A_24_P160104	*TUBA8*	NM_018943						1		1	19
A_24_P318656^*^	*ITGB3*	NM_000212						1		1	
A_24_P65373^**^	*ITGA2B*	NM_000419						1		1	
A_32_P168342^^^	*ENST00000299289*	ENST00000299289						1		1	
A_32_P168349^^^	*ENST00000299289*	ENST00000299289						1		1	
A_32_P196142	*THC2400010*	THC2400010						1		1	
A_32_P199824	*THC2317149*	THC2317149						1		1	
A_32_P209230	*CITED4*	NM_133467						1		1	
A_24_P194313	*C21orf66*	BC062992						1		1	
A_32_P81173	*USP34*	AL050376						1		1	
A_23_P39237	*ZFP36*	NM_003407				1				1	**2**
A_23_P90172	*PPP1R15A*	NM_014330				1				1	
A_32_P115749	*CD104030*	CD104030				1	1				**2**
A_32_P206308	*THC2400121*	THC2400121				1	1				
											**23**

^*^Two Agilent duplicate spots for *ITGB3* gene; ^**^Two Agilent duplicate spots for *ITGA2B* gene. ^^^Two Agilent duplicate spots for ENST00000299289.

We also used more relaxed conditions (*p*-value ≤ 0.05 plus ≥ 1.3-fold change) in order to obtain a larger list of candidate genes. Under these relaxed conditions, a total of 997 transcripts from four groups were identified. Top 20 down- or up-regualted DEGs are provided in Supplementary Table 9 for each group. Consistently, more genes were differentially expressed in responders than in non-responders at both Day 2 (RD2: 549 genes vs. ND2: 66 genes) and at one month post-ECP (RM1: 472 genes vs. NM1: 95 genes) (Figure 2). RM1 group had 472 DEGs, and there were twice as many down-regulated DEGs (313) as up-regulated DEGs (159).

These results suggest that transcriptional changes after ECP in responders are larger than in non-responders, and the down-regulation of gene expression is the dominant and lasting effect.

### Functional associations of DEGs

**Figure 3:**
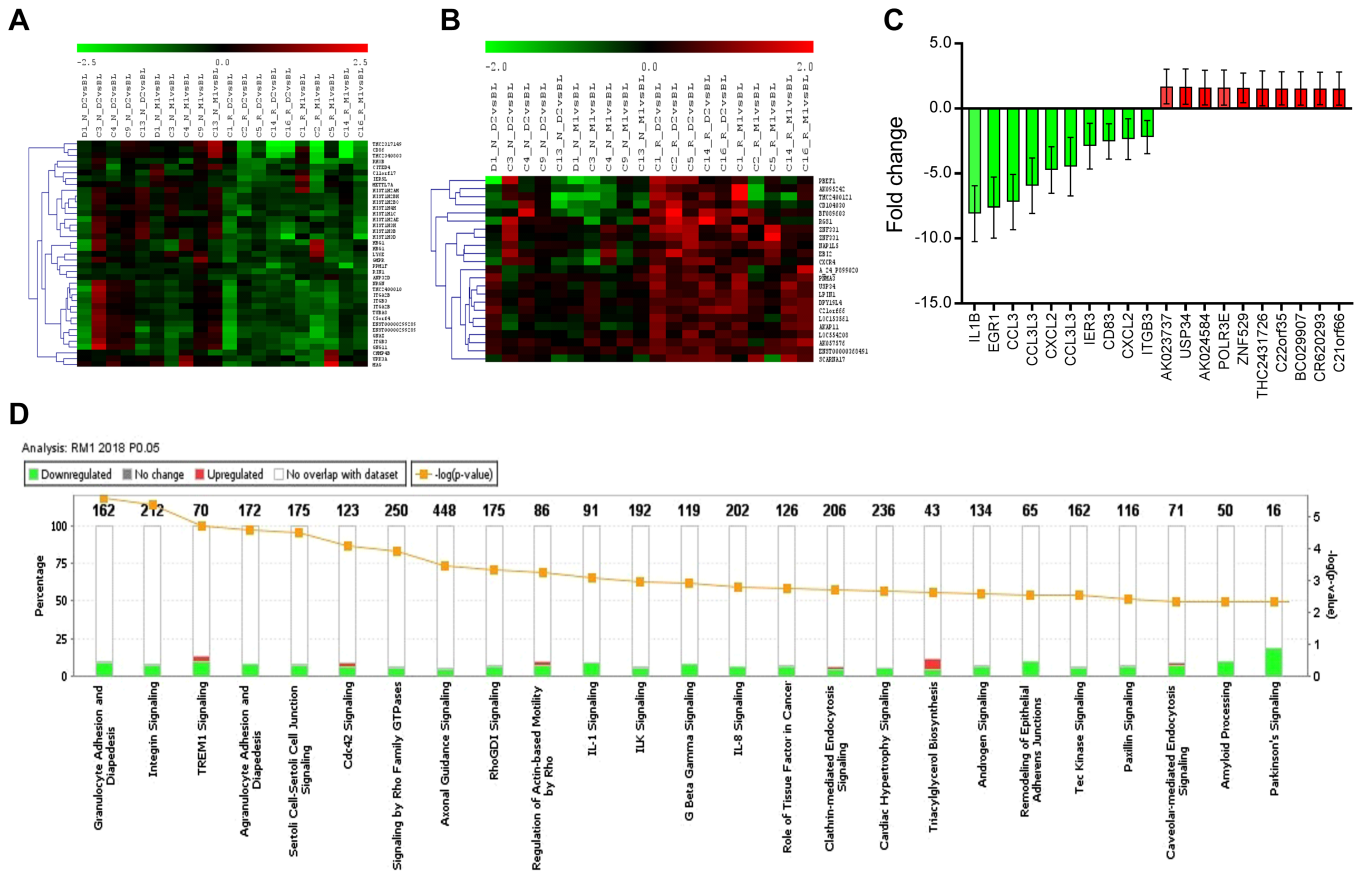
Hierarchically clustered heatmap of differentially expressed genes (DEG), top DEGs, and canonical biological pathways enriched in DEG in RM1. DEGs with *p* ≤ 0.05 and fold change ≥1.3 were uploaded for Ingenuity Pathway Analysis. A core analysis with default parameters was conducted, and the top regulated DEGs and top canonical biological pathways were identified. (**A**) The hierarchically clustered heatmap of down-regulated DEGs in clinically-responsive patients at Day 2 post-ECP (RD2, *n* = 5); (**B**) The hierarchically clustered heatmap of up-regulated DEGs in RD2 (*n* = 5); (**C**) Top 20 dysregulated DEGs in clinically-responsive patients at one month post-ECP (RM1, *n* = 5)); data are represented as mean ± SD; and (**D**) Top 25 canonical biological pathways enriched in DEGs by IPA in RM1.

Next, we performed hierarchical clustering analysis (HCL) for all DEGs (*p* ≤ 0.05 and ≥1.3-fold change) from four groups (RD2, RM1, ND2, and NM1). The clustered DEGs from two responder groups, RD2 and RM1, show a more consistent downregulation or upregulation, respectively, while the expression profiles for two non-responder groups, ND2 and NM1, are more variable. Exemplary hierarchical clustering heat-maps for down-regulated and up-regulated DEGs from RD2 group are showed in Figure 3A and 3B.

In order to define the top DEGs and their functional associations, we then performed a core analysis by Ingenuity Pathway Analysis (IPA) for four groups. The organismal injury and abnormalities are the top diseases and bio functional associations among all four groups, and followed by hematological diseases in 3 of 4 groups (RD2, RM1, and ND2). The core analysis of DEGs from RM1 group indicate that the top five related molecular and cellular functions were: cellular development (151 genes), cellular growth and proliferation (143 genes), cell-to-cell signaling and interaction (75 genes), cellular function and maintenance (153 genes), and cell death and survival (159 genes). The core analysis of DEGs from RM1 indicate that the top five related physiological system development and functions were: hematological system development and function (133 genes), hematopoiesis (83 genes), tissue development (103 genes), and tissue morphology (114 genes), and immune cell trafficking (64 genes). The top five related diseases were: hematological disease (144 genes), connective tissue diseases (82 genes), organismal injury and abnormalities (283 genes), hereditary disorders (28 genes), and infectious disease (97 genes).

The top down-regulated DEGs in RM1 group are related to functions of platelets, immune and/or stress responses, and chromatin remodeling, with *IL1B*, *EGR1*, *CCL3*, *CCL3L3*, and *CXCL2* at the top of the list as shown in Figure 3C. The top up-regulated genes are *AHSA2P*, *POLR3E*, *ZNF529*, *MIAT*, and *PAXBP1,* which are related to functions of the nucleolus. In addition to more down-regulated DEGs, there was a big range of 1.3 to 8.1-fold decrease in down-regulation of DEGs compared to only 1.3 to 1.7-fold increase in up-regulated DEGs.

These results suggest that ECP exerts a comprehensive effect on multiple molecular and cellular functions, and primarily inhibitory effects may underlie the effectiveness of ECP in clinically-responsive patients.

### Canonical biological pathways

To further define the biological pathways related to transcriptome response, a pathway enrichment analysis using IPA was performed for all 4 groups (RD2, RM1, ND2, and NM1). The top canonical biological pathways affected were different between responder groups (RM1 and RD2) and non-responder groups (NM1 and ND2), listed in Table 3. The top canonical pathways were also different between the early time points (RD2 and ND2) and the later time points (RM1 and NM1).

**Table 3:** The top 5 canonical biology pathways and related differentially expressed genes (DEGs) in responder groups (RM1 and RD2) and non-responder groups (NM1 and ND2)

	Canonical pathways	Downregulated DEGs	Upregulated DEGs
RM1	Granulocyte Adhesion and Diapedesis	14/162 (9%): *CSF3R, ICAM1, PPBP, ITGA5, CXCL5, SDC4, CCL3, ITGB3, GNAI2, CLDN5, CCL3L3, IL1B, CXCL1, CXCL2*	1/162 (1%): *ITGB1*
	Integrin Signaling	15/212 (7%): *ITGA2B, MAP3K11, ITGA5, MYLK, ITGB3, MYL9, PARVB, AKT1, RHOB, CAPN1, ACTN4, CTTN, ARPC4, ACTN1, ITGB5*	2/212 (1%):*ITGB1, PPP1R12A*
	TREM1 Signaling	7/70 (10%): *ICAM1, AKT1, NLRP12, ITGA5, IL1B, CD83, CCL3*	2/70 (3%): *ITGB1, NLRC3*
	Agranulocyte Adhesion and Diapedesis	13/172 (8%): *ICAM1, PPBP, ITGA5, CXCL5, SDC4, CCL3, GNAI2, MYL9, CLDN5, CCL3L3, IL1B, CXCL1, CXCL2*	1/172 (1%):*ITGB1*
	Sertoli Cell-Sertoli Cell Junction Signaling	13/175 (7%): *TUBB1, MAP3K11, TUBA4A, ITGA5, MAPK14, AKT1, PRKAR2B, CLDN5, TUBA8, SPTB, PRKACA, ACTN4, ACTN1*	1/175 (1%): *ITGB1*
RD2	Opioid Signaling Pathway	15/239 (6%): *AP2M1, AP2A1, CAMK1, AP1B1, GNAI2, CALM1 (includes others), PRKAR2B, GNG11, AKT1, CACNA1B, PRKACA, PNOC, RGS14, RPS6KA4, FGR*	3/239 (1%): *RGS1, ATF2, CTNNB1*
	Transcriptional Regulatory Network	6/53 (11%): *HIST1H4F, HIST2H4B, HIST1H4I, OTX1, HNF4A, HIST1H4H*	2/53 (4%): *RIF1, SMARCAD1*
	Lipid Antigen Presentation by CD1	5/19 (26%): *AP2A1, AP2M1, PSAP, CANX, AP1B1*	0/19 (0%)
	Amyloid Processing	6/50 (12%): *PRKAR2B, AKT1, CAPN1, PRKACA, APP, PSEN1*	1/50 (2%): *CSNK1D*
	DNA Methylation and Transcriptional Repression	4/33 (12%): *HIST1H4F, HIST2H4B, HIST1H4I, HIST1H4H*	1/33 (3%): *MECP2*
NM1	Polyamine Regulation	2/21 (10%): *SAT1, CTNNB1*	0/21 (0%)
	PRPP Biosynthesis I	1/3 (33%): *PRPS1*	0/3 (0%)
	Spermine and Spermidine Degradation I	1/4 (25%): *SAT1*	0/4 (0%)
	Unfolded protein response	1/55 (2%): *PPP1R15A*	1/55 (2%): *BCL2*
	Sirtuin Signaling	4/277 (1%): *PFKFB3, MT-CYB, GABARAPL1, ATG16L2*	0/277 (0%)
ND2	Eicosanoid Signaling	3/62 (5%): *PLA2G16, PLB1, PTGDS*	0/62 (0%)
	Phospholipases	2/56 (4%): *PLA2G16, PLB1*	0/56 (0%)
	Role of Macrophages	4/306 (1%): *IRAK3, CEBPB, TCF7L2, FCGR3A/FCGR3B*	0/306 (0%)
	Glycoaminoglycan-protein Linkage Region Biosynthesis	1/7 (14%): *B3GAT1*	0/7 (0%)
	Regulation of the Epithelial-Mesenchymal Transition Pathway	2/189 (1%): *ZEB2, TCF7L2*	1/189 (1%): *TWIST1*

Abbreviations: DEGs: differentially expressed genes; ECP: extracorporeal photopheresis; BL: baseline; ND2: samples collected after treatment at Day 2 from patients resistant to ECP; NM1: samples collected after treatment at 1 month post-ECP from patients resistant to ECP; RD2: samples collected after treatment at Day 2 from patients responsive to ECP; RM1: samples collected after treatment at 1 month post-ECP from patients responsive to ECP.

The stacked bar chart in Figure 3D shows the top 25 canonical pathways for RM1 group based on DEGs. The top pathways found to be affected were: 1) granulocyte adhesion and diapedesis; 2) integrin signaling; 3) triggering receptor expressed on myeloid cells 1 (TREM1) signaling; and 4) agranulocyte (lymphocyte, monocyte and macrophage) adhesion and diapedesis. These affected pathways were closely related to cell attachment, adhesion and diapedesis, with the integrin signaling pathway at the top. There were 17 genes or molecules in the integrin signaling differentially expressed, with 15 genes down-regulated and two genes up-regulated. Out of 5 integrin genes, *ITGA2B,* *ITGA5*, *ITGB3*, and *ITGB5* were down-regulated, and *ITGB1* was up-regulated. Down-regulation of *ITGA2B* and *ITGB3* was seen in both RD2 and RM1groups, and duplicate Agilent spots for these two genes were also consistently down-regulated (Table 2, Supplementary Table 2, and Supplementary Table 4).

These results suggest that multiple biological pathways are affected at one month post-ECP treatment in clinically-responsive patients, with notable modulation of cell attachment, adhesion and diapedesis.

### Transcription regulators and other upstream regulators

**Figure 4:**
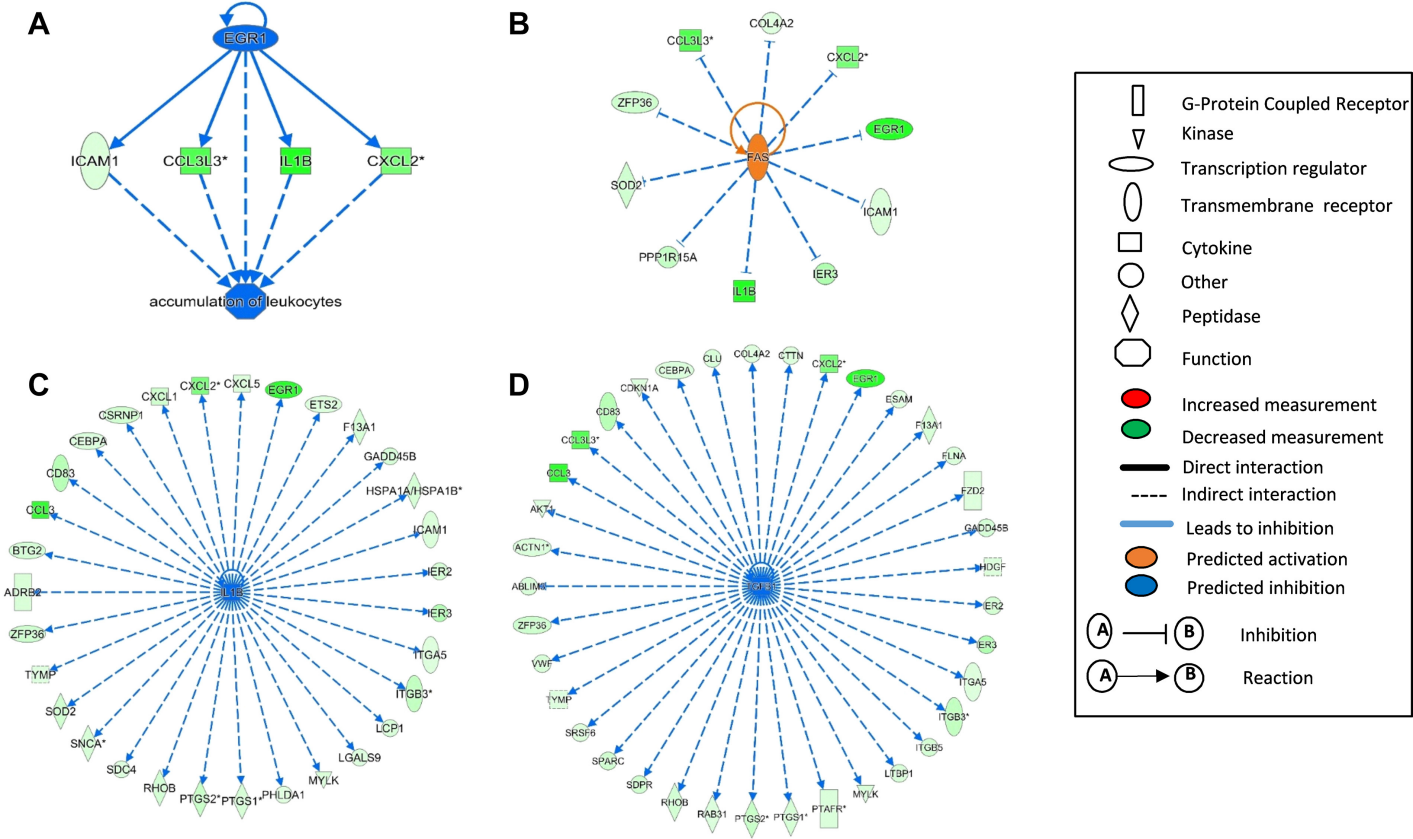
Upstream regulators affected by ECP enriched in differentially expressed genes (DEG) in RM1. The upstream regulators *EGR1* (**A**), *FAS* (**B**), *IL1B* (**C**), and *TGFB1* (**D**) were identified from differentially expressed genes (DEGs) by Ingenuity Pathway Analysis (IPA) in clinically-responsive patients at one month post-ECP (RM1, *n* = 5). The full symbol legends are included in the figure.

We further explored the involvement of upstream regulators affected by ECP. There were three transcription factors (*EGR1*, *ZFP36*, and *KLF6*) among down-regulated DEGs in RM1 group, with *EGR1* having a 7.6-fold down-regulation (Table 4). *EGR1* or early growth response 1 is a master regulator of hematopoietic differentiation, and studies demonstrated that the dysregulation of EGR1 is involved in hematologic malignancies such as chronic lymphocytic leukemia and B cell lymphoma [21]. The down-regulation of *EGR1* is predicted to decrease the transcription of *ICAM1*, *CCL3L3*, *IL1B* and *CXCL2*, and could lead to an inhibition of the accumulation of leukocytes (Figure 4A). Interestingly, *IL1B*, *CCL3L3*, *CDKN1A*, and *PTGS2* were all down-regulated by three transcription factors. Thus, *EGR1*, *ZFP36*, and *KLF6* are likely involved in the regulation of the expression of these downstream genes.

**Table 4:** Transcription factors affected by ECP

Upstream regulator	Fold change	Molecule type	Predicted activation state	Target molecules in dataset	Mechanistic network
***EGR1***	–7.624	transcription regulator	Inhibited	*CCL3L3, CDKN1A, CLU, CXCL2, EGR1, GADD45B, ICAM1, IL1B, PTGS2, SOD2*	147 (21)
***ZFP36***	–1.876	transcription regulator	Activated	*CCL3L3, CDKN1A, ICAM1, IL1B, LATS2, PTGS2*	75 (14)
***KLF6***	–1.563	transcription regulator	Inhibited	*CCL3L3, CDKN1A, CXCL2, IL1B, PMAIP1, PTGS2, SHH*	82 (13)

Other upstream analyses suggest that *FAS*, *IL1B*, and *TGFB1* are the top upstream regulators, with *TGFB1* and *IL1B* predicted to cause inhibition and *FAS* predicted to cause activation. Interestingly, dysregulation of these three molecules and related networks are known to be involved in the pathogenesis of CTCL [4, 8, 22]. Activation of *FAS* not only could enhance activation-induced cell death (AICD), but has also been predicted to inhibit many transcripts including *EGR1* and its down-stream molecules (Figure 4B). *IL1B*, the top down-regulated DEG in RM1 group (–8.088 fold change), could contribute to transcriptome alteration for at least 30 molecules as shown in Figure 4C. *IL1B* is a master upstream regulator for many molecules involved in granulocyte/agranulocyte adhesion and diapedesis, dendritic cell differentiation, regulation of cytokine production, and *NF-kB* signaling. Finally, among the predicted molecules inhibited by *TGFB1*, multiple cytokines, transmembrane receptors, peptidases, G-protein coupled protein, and kinases, are also affected by *IL1B* (Figure 4D).

### Comparison analysis between different groups

**Figure 5:**
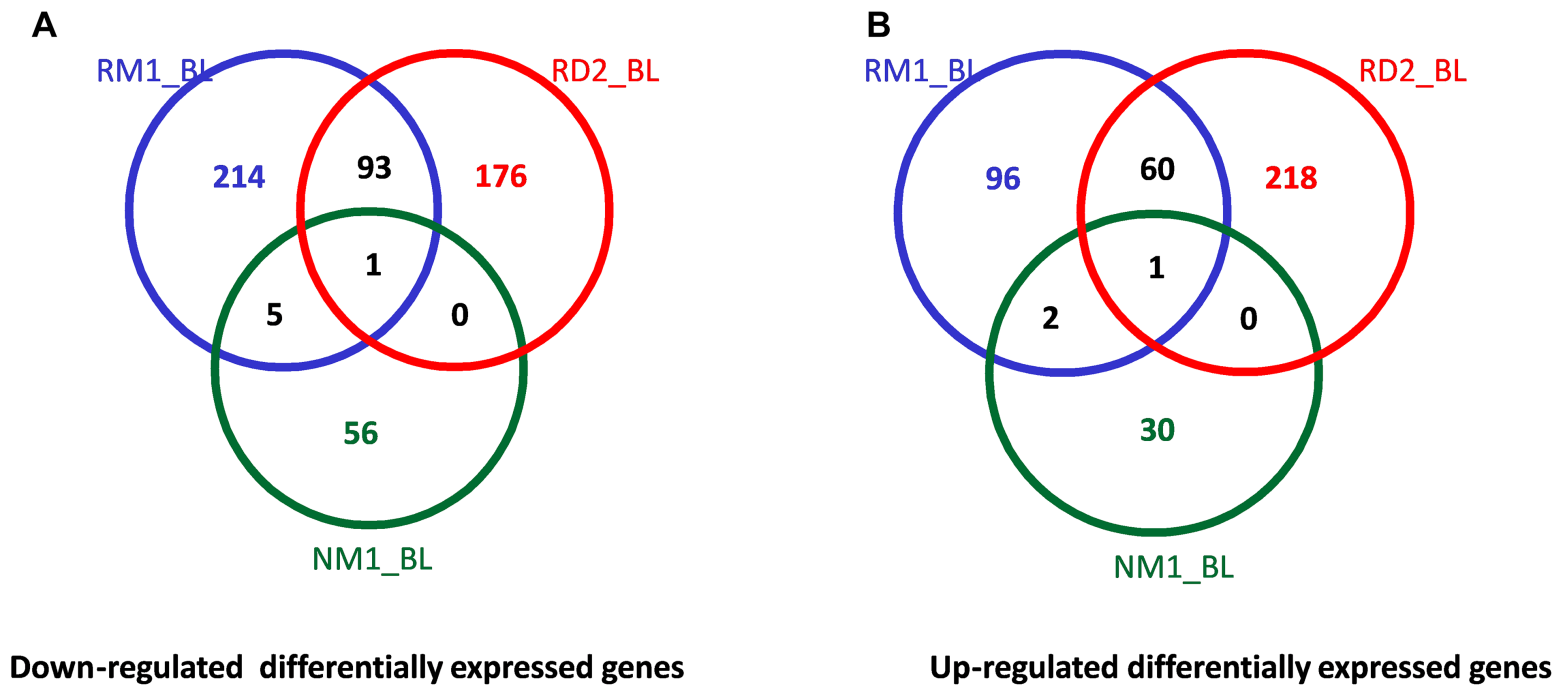
Venn diagrams of the cross comparison of DEGs between different groups. The numbers of downregulated (**A**) and upregulated (**B**) differentially expressed genes (DEGs, *p* ≤ 0.05, fold change ≥1.3) were cross compared between responders at Day 2 (RD2_BL, *n* = 5) and one month post-ECP (RM1_BL, *n* = 5) and non-responders at one month post-ECP (NM1_BL, *n* = 5).

As mentioned previously, with the stricter cutoff of *p* ≤ 0.05 and fold change ≥1.5, there were 19 DEGs observed in two responder groups, RD2 and RM1, but few DEGs in common to the non-responder groups (ND2 or NM1) (Table 2). Consistently, with a cutoff of *p* ≤ 0.05 and relaxed fold change ≥1.3, there were 94 genes downregulated in both RD2 and RM1 groups while only 6 genes were found in common between RM1 and NM1 groups (Figure 5A). Similarly, 61 genes were upregulated in both RD2 and RM1 group, while only 3 genes were in common between RM1 group and the NM1 group (Figure 5B).

**Figure 6:**
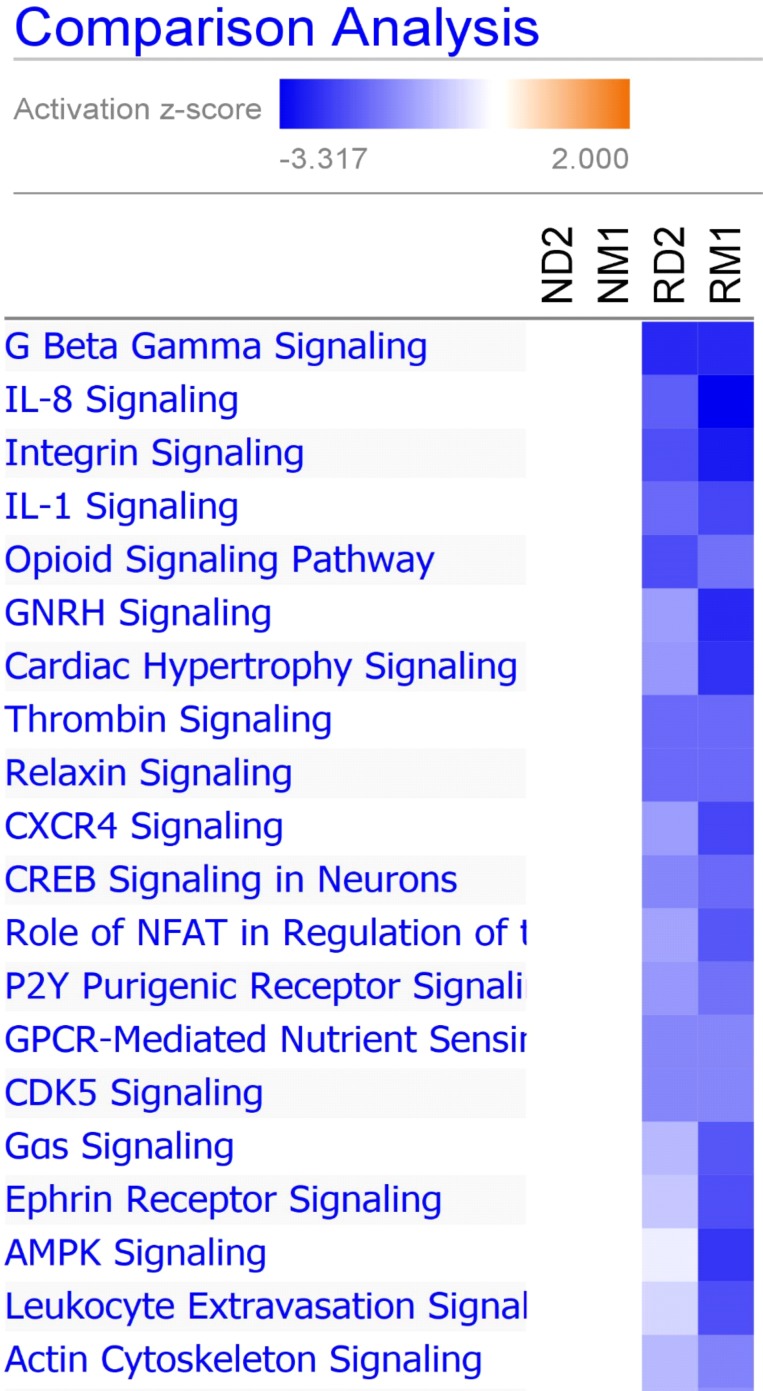
Canonical pathway comparison between different groups. Ingenuity Pathway Analysis comparison analysis tool was used to generate a heat map depicting predicted activation or inhibition of canonical pathways. Blue represents predicted inhibition, and red represents predicted activation. Increasing significance, or activation z-score, is represented by increasing intensity of color. Displayed are top 20 canonical pathways comparing responders at Day 2 (RD2) and 1 month (RM1) to non-responders at Day 2 (ND2) and 1 month (NM1) post-ECP.

To identify the differences in canonical biological pathways between responders and non-responders, we further performed comparison analysis using IPA for four groups (RD2, RM1, ND2, and NM1). As shown in Figure 6, there were multiple pathways shared between two responder groups (RD2 and RM1), but few or none were shared with non-responder groups (ND2 and NM1). The top overlapped pathways between RD2 and RM1 groups are G-beta gamma signaling, IL-8 signaling, integrin signaling, and IL-1 signaling pathways.

These results indicate that that transcriptional changes, DEGs and their related pathways, following ECP therapy, differ between responders and non-responders.

### Confirmation of *IL1B, EGR1,* and *ITGB3* expression

**Figure 7:**
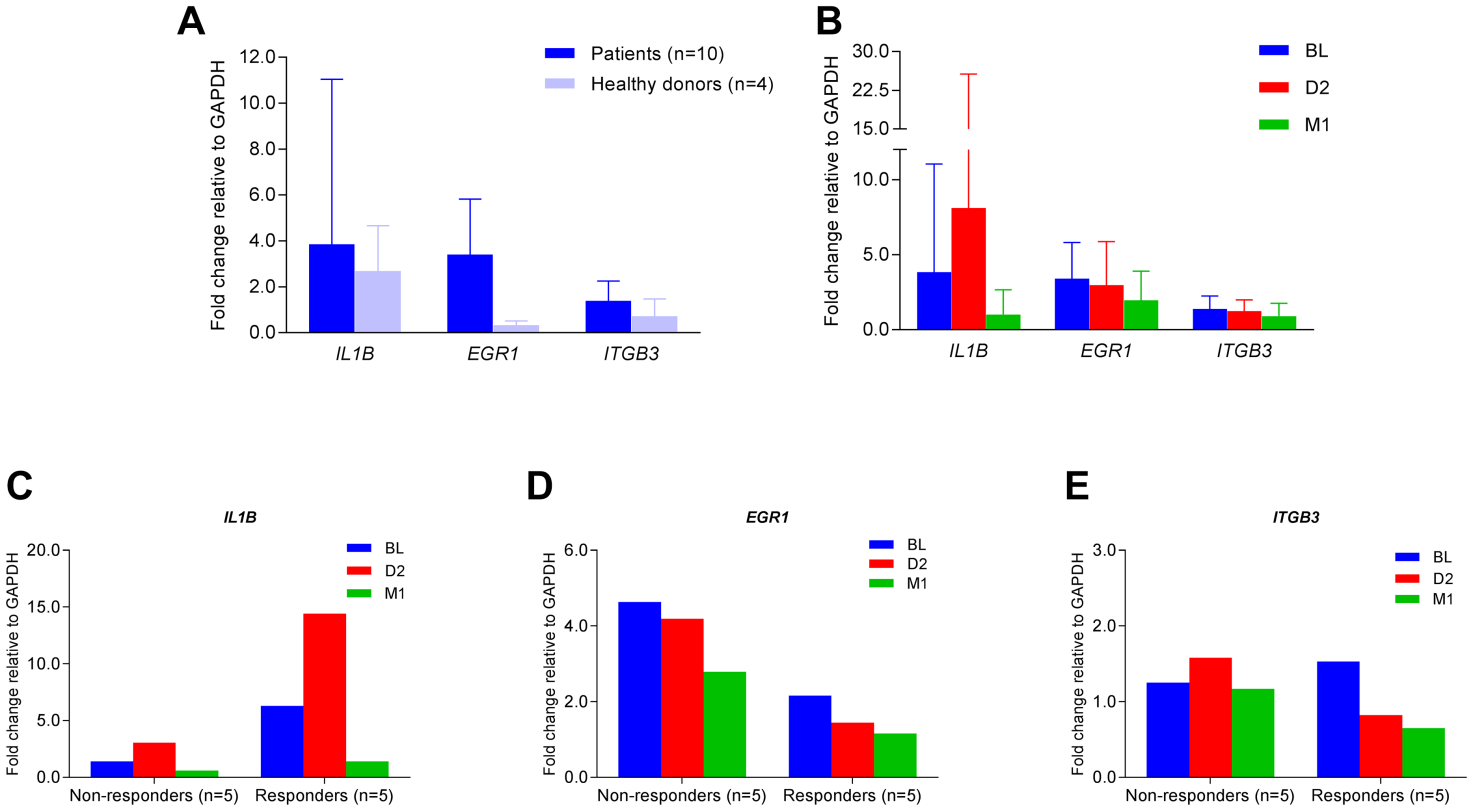
Quantitative real-time PCR for *IL1B*, *EGR1*, and *ITGB3* mRNA expression. Quantitative real-time PCR was conducted to assess the relative levels of mRNA expression of *IL1B*, *EGR1*, and *ITGB3* with pre-formulated TaqMan primers and probes. Relative fold changes were calculated and normalized to GAPDH. (**A**) Fold changes of *IL1B*, *EGR1*, and *ITGB3* mRNA in PBMCs of patients (*n* = 10) and healthy donors (*n* = 4); data are represented as mean ± SD; (**B**) Fold changes of *IL1B*, *EGR1*, and *ITGB3* mRNA at BL, D2 and M1 in PBMCs of patients (*n* = 10); data are represented as mean ± SD; (**C**) Fold changes of *IL1B* mRNA at BL, D2 and M1 in PBMCs of clinically responsive patients (*n* = 5) versus non-responders (*n* = 5); (**D**) Fold changes of *EGR1* mRNA at BL, D2 and M1 in PBMCs of clinically responsive patients (*n* = 5) versus non-responders (*n* = 5); (**E**) Fold changes of *ITGB3* mRNA at BL, D2 and M1 in PBMCs of clinically responsive patients (*n* = 5) versus non-responders (*n* = 5).

To confirm our microarray findings, we employed real-time quantitative PCR (qPCR) to examine mRNA expression of *IL1B*, *EGR1*, and *ITGB3* in these L-CTCL patients. Higher expression of *IL1B*, *EGR1*, and *ITGB3* was present in patients (*n* = 10) compared to healthy donors (*n* = 4) (Figure 7A). Overall, three genes were down-regulated at one month post-ECP (Figure 7B). Expression of *IL1B* was initially increased at D2 but was decreased at M1 post-ECP in both responders and non-responders (Figure 7C). Expression of *EGR1* was decreased at D2 and M1 post-ECP in both responders and non-responders (Figure 7D). Expression of *ITGB3* was decreased at D2 and M1 post-ECP in responders, but not in non-responders (Figure 7E). Together, these results confirm our microarray data.

## DISCUSSION

In this cohort of L-CTCL patients receiving ECP therapy, we identified numerous transcriptional changes in the peripheral blood at Day 2 and 1 month post-ECP treatment in clinically-responsive patients. We found that downregulated genes accounted for two thirds of all differentially expressed genes, and confirmed a primarily inhibitory effect of ECP on the transcriptome of PBMCs in these patients. We also found that multiple biological pathways were affected by ECP. The most common pathways include the integrin signaling, granulocyte and agranulocyte adhesion and diapedesis, and IL-1 signaling. Our results indicate that the modulation of cell adhesion and diapedesis and suppression of IL1β induced inflammation underlie ECP efficacy in L-CTCL patients.

Until recently, induction of apoptotic cell death of malignant T cells and immune modulation were thought to be the main mechanisms of action of ECP in patients with L-CTCL [19]. Our data support the induction of activation-induced cell death by ECP. For example, *FAS* activation by ECP was identified as an upstream regulator, and many genes regulated by *FAS* may contribute to the apoptosis. *IL1B* and *TGFB1* inhibition also support immunomodulatory effects of ECP in CTCL.

With the power of a 44K microarray, we were able to fully profile the transcriptional changes after ECP and identified additional transcripts/molecules and related biological pathways. We found that not only regulation of immune and/or stress responses, but also modulation of platelet functions and the nucleolus and chromatin remodeling, contribute to the action of ECP. Multiple canonical biological pathways are affected by ECP in patients responsive to therapy compared to patients resistant to therapy. Most important, a unique transcriptome modification by ECP is related to biological pathways involved in cell attachment, adhesion, and diapedesis, including the integrin signaling pathway.

Recently, multiple integrin members have been implicated in tumor initiation and progression [23]. Integrins play a key role in regulating T-cell migration [11]. Grabbe, et al. reported that β2 integrins are required for skin homing of primed T cells but not for the priming naive T cells [24]. The β2 integrins enable lymphocytes to attach firmly to endothelial cells at sites of infection and migrate out of the bloodstream into the infected site. In addition, all-trans-retinoic acid (ATRA) and bexarotene were able to decrease β2 integrin expression in a CTCL cell line (Hut78 cells) but increased β1 integrin expression. Both ATRA and bexarotene also increased β7-dependent adhesion [25]. Our data are consistent with the action of retinoid since there was an increase in β1 integrin and a decrease in β2 integrin expression after ECP therapy, however no changes in β7 integrin expression were seen.

The induction of monocyte to dendritic cell (DC) differentiation by ECP in L-CTCL patients has been reported by us and others [17, 18]. However, the signaling pathways underlying this process are not fully understood. Recently, Gonzalez, et al. reported that monocytes passed through protein-modified ECP plates adhered transiently to plasma proteins, including fibronectin, and activated signaling pathways that initiate the monocyte-to-DC conversion [20]. Fibronectin and other plasma proteins were able to act through cell adhesion via αVβ3 and α5β1 integrin signaling to drive monocyte-to-DC differentiation [20]. Our findings suggest that ECP modulates the integrin signaling pathway by regulating β1 and β2 integrins affecting both T-cell skin homing and monocyte to DC differentiation. Integrins are critical players in numerous cancers, and could also be pertinent therapeutic targets [23, 26].

Recent studies indicate that inflammation mediated by IL-1β may have a major role in cancer invasiveness, progression, and metastases [27, 28]. Wu, *et al.* reported an inflammation-dependent mouse model of skin T-cell lymphoma tumorigenesis [29]. They found that the application of dinitrofluorobenzene (DNFB) activated IL-1β in the mouse skin and recombinant IL-1β could partially replace DNFB treatment as an enabler of tumor growth in their model [29]. Our findings suggest that ECP may suppress the IL-1 signaling pathway by downregulating IL-1β which could affect cell adhesion, DC differentiation, regulation of cytokine production, and NF-kB signaling. Multiple studies suggest that IL-1β may be valuable target for both the prevention and treatment of cancer and cancer therapy–related complications [27, 28]. Clinical studies targeting IL-1β have already been performed in both solid tumors and hematological malignancies [27, 28].

This small pilot study has a few weaknesses. Although ECP can produce a complete response in 14%-33% of patients with CTCL [13–15], unfortunately, none of our clinically-responsive patients achieved complete responses. Our patients had high tumor burdens and the sample size was limited to five patients in each group. It would be very interesting to specifically compare the molecular changes in patients with complete response to non-responders. Total RNA samples used in this study were extracted from peripheral blood mononuclear cells (PBMCs) in patients with L-CTCL. PBMCs are composed of a mixture of lymphocytes, monocytes, NK cells and these could contribute the transcriptional changes following ECP therapy. We have conducted parallel flow cytometry analysis for Sézary cells, T-cell subsets, and dendritic cells in the peripheral blood in these patients [18, 19]. Recently, new analysis methods are being developed to allow scientists to further cluster transcripts into different blood and immune cell types [30]. Furthermore, profiling transcriptional changes in a single cell population or even at a single cell level are now possible and may give clearer data or insights. Since our study cohort was heterogeneous and included patients who underwent ECP alone and with combined immunomodulatory therapy, we cannot fully attribute clinical responses to ECP. It is noteworthy though that combinational therapies were added at 3 months after ECP, whereas our samples were collected at baseline and earlier time points, Day 2, and 1 month after ECP. In spite of its small sample size and stated weaknesses, our findings provide valuable results, and future studies are warranted to further explore these insights.

**Figure 8:**
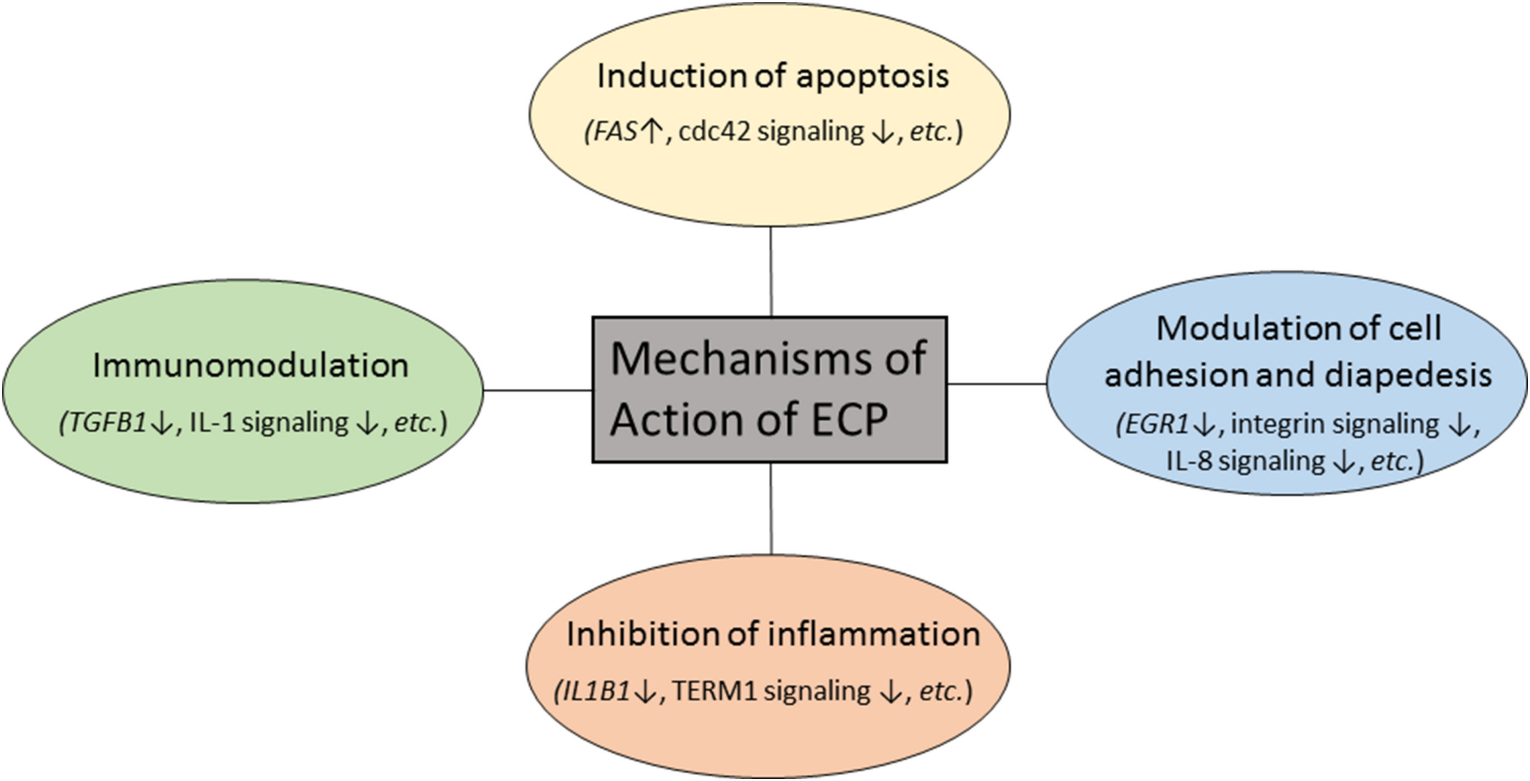
Proposed mechanisms of action of extracorporeal photopheresis in patients with L-CTCL.

In summary, we used microarrays and pathway analysis to identify key transcriptional changes over a course of ECP treatment in ten L-CTCL patients. In addition to the previous known mechanisms of action of ECP, we identified new genes and biological pathways that may be relevant to clinical responses to ECP (Figure 8). These findings may help us to better understand mechanisms of action of ECP therapy in L-CTCL patients and pathogenesis of L-CTCL. Our findings may also be applicable to other diseases which benefit from ECP treatment such as GVHD and scleroderma. Our findings provide hints for identifying new potential therapeutic targets for L-CTCL patients.

## MATERIALS AND METHODS

### Patients and sample collection

Ten patients with L-CTCL from our previous study [18, 19] included five clinically-responsive responders (R) and five clinically-resistant non-responders (NR) (Table 1). The study was conducted according to the Declaration of Helsinki and approved by the Institutional Review Board of the University of Texas MD Anderson Cancer Center (MDACC). All patients signed informed consent and were treated with the THERAKOS UVAR XTS photopheresis system (Mallinckrodt Pharmaceuticals, Bedminster, NJ, USA) over 2 consecutive days every 2–4 weeks per cycle. ECP was given as a monotherapy to all patients during the first 3 months. Bexarotene and/or interferon alpha (IFNα) were added if patients were not responding to ECP at 3 months. Fresh peripheral blood samples were collected before ECP treatment at baseline (BL) and after treatment at Day 2 (D2), 1 month (M1), 3 months (M3), and 6 months (M6). Clinical responses were assessed at 6 months post-therapy by changes in skin disease using the modified severity-weighted assessment tool (mSWAT) and by changes in circulating CD4^+^CD26^–^ malignant T cells, as previously described [18, 19].

### Flow cytometry analysis of T-cell subsets and dendritic cell subsets

Peripheral blood mononuclear cells (PBMC) were isolated by Ficoll density gradient centrifugation. PBMCs from BL, M1, M3, and M6 were stained with fluorescence conjugated anti-human CD3, CD4, CD8, CD26, or/and CD25 monoclonal antibodies, and analyzed by flow cytometry for different T-cell subsets as previously reported [19] [31, 32]. PBMCs were also stained with anti-human Lin, HLA-DR, CD11c, and CD123 for analysis of Lin^−^HLA-DR^+^CD11c^+^ myeloid dendritic cells (mDCs) and Lin^−^HLA-DR^+^CD123^+^ plasmacytoid dendritic cells (pDCs), as previously described [18].

### Total RNA extraction and the agilent whole human genome microarray

For the microarray assays, we chose two time points, Day 2 (D2) and 1 month (M1) following ECP treatment, for assessment of early and late response genes compared to baseline. D2 samples were taken 24 hours immediately after the 1st ECP treatment, and M1 samples were taken after 1 or 2 cycles of treatment and just before a new cycle of ECP treatment began. Total RNA was extracted using the RNeasy Mini Kit (Cat No.74104, Qiagen, USA) from PBMCs. Quantity and quality of total RNA was determined by NanoDrop™ 1000 Spectrophotometer (Thermo Scientific, CA, USA) and Agilent BioAnalyzer 2100 (Agilent, Santa Clara, CA, USA). Microarrays were performed by the MDACC Genomics Core Facility. In brief, for each hybridization, 500 ng of Cyanine 5 (Cy5) labeled cRNA (the treated sample) and 500 ng of Cyanine 3 (Cy3) labeled cRNA (the corresponding baseline sample) were mixed, fragmented and co-hybridized at 65°C for 18 hours to a Whole Human genome Oligo Microarray (Agilent 4 × 44 K product G4112F, Santa Clara, CA, USA). The microarray images were scanned using an Agilent microarray scanner. Feature extraction software (Agilent, Santa Clara, CA, USA) was used to assess fluorescent hybridization signals. Twenty total RNA samples were used for microarray experiments (Supplementary Table 1).

### Bioinformatic analysis

Bioinformatic analysis of microarray data was done by Bioinformatics Service, Miltenyi Biotec GmbH (Bergisch Gladbach, Germany) using R/Bioconductor and software packages therein. First, signal intensities of the Cy3 and Cy5 channels were background corrected and normalized for dye effects by LOESS normalization, followed by adjustment of intensity differences between the arrays by quantile normalization. Then, the ratio data were log2-transformed. Student *t*-test (*P* value ≤ 0.05) was used to identify differentially expressed genes (DEGs) between baseline (BL) and Day 2 (D2) or 1 month (1M) after therapy. The fold changes of expression signals between D2 or 1M to their corresponding BL were calculated from the normalized values. In addition to a *p*-value ≤ 0.05, genes selected as reliable candidates were required to show at least 1.5-fold average expression difference. For the comparisons to the non-responder group, none or only very few reporters were identified as candidate genes with differential expression using these selection criteria. A better overview on affected functions was obtained with more relaxed conditions which resulted in a larger lists of candidate genes. These relaxed conditions were defined by a *p*-value ≤ 0.05 and at least 1.3-fold expression difference relative to the corresponding BL. Hierarchical clustering was performed with Euclidean distance using DEGs (16). All data are deposited in the Gene Expression Omnibus (GEO) database (accession number GSE114891).

### Ingenuity pathway analysis

The differentially expressed gene lists generated from microarray analysis which met the relaxed criterion above were uploaded for Ingenuity Pathway Analysis (IPA, Ingenuity Systems, Redwood City, CA, USA) (17). A core analysis with default parameters was conducted, and the top regulated DEGs, top canonical biological pathways, and top upstream regulators were identified. The network analysis was also used to display an interactive graphical representation of the interrelationships between genes. DEGs from responders (RD2, RM1) and non-responders (ND2, NM1) were cross compared using IPA comparison analysis.

### Quantitative real-time PCR for *IL1B, EGR1,* and *ITGB3* mRNA expression

First strand cDNA was synthesized using 400 ng of total RNA, from the same batch used in the microarray, using oligo (dT) 12–18 primer and Superscript IV reverse transcriptase (Life Technologies Inc., Gaithersburg, MD, USA). Pre-formulated TaqMan primers and probes for *IL1B* (Hs 01555410_m1), *EGR1* (Hs 00152928_m1), and *ITGB3* (Hs 01001469_m1) were used. *GAPDH* (Hs99999905_m1) was used as an endogenous control gene. Quantitative PCR was performed with the Applied Biosystems™ StepOnePlus™ Real-Time PCR System using the default manufacturer protocol (Applied Biosystems, Foster City, CA, USA). Relative levels of gene expression were quantitated based on Ct values and then normalized to *GAPDH*. Relative fold changes were calculated as previously described [33].

## SUPPLEMENTARY MATERIALS



## Abbreviations

CTCL: cutaneous T-cell lymphoma
SS: Sézary syndrome
MF: mycosis fungoides
ECP: extracorporeal photopheresis
PBMC: peripheral blood mononuclear cell
IPA: Ingenuity Pathway Analysis
DEG: differentially expressed gene
*IL1B*: interleukin 1 beta
*EGR1*: early growth response 1
*ITGB3*: integrin beta 3
BL: baseline
ND2: Day 2 post-ECP, non-responders
NM1: 1 month post-ECP, non-responders
RD2: Day 2 post-ECP, responders
RM1: 1 month post-ECP, responders

## Notes

**Editorial note**

This paper has been accepted based in part on peer-review conducted by another journal and the authors’ response and revisions as well as expedited peer-review in Oncotarget.
