# Correction: Blood transcriptional profiling reveals IL-1 and integrin signaling pathways associated with clinical response to extracorporeal photopheresis in patients with leukemic cutaneous T-cell lymphoma

**DOI:** 10.18632/oncotarget.27197

**Published:** 2019-09-10

**Authors:** Zuolin Ying, Lisa Shiue, Katherine Park, Jutta Kollet, Pedram Bijani, Meghali Goswami, Madeleine Duvic, Xiao Ni

**Affiliations:** ^1^ Department of Dermatology, The University of Texas MD Anderson Cancer Center, Houston, TX 77030, USA; ^2^ Bioinformatics, Miltenyi Biotec GmbH, Bergisch Gladbach, 51429, Germany

**This article has been corrected:** The #2 affiliation information has been updated. The proper institution name is shown below:

^2^Bioinformatics, Miltenyi Biotec GmbH, Bergisch Gladbach, 51429, Germany

Original article: Oncotarget. 2019; 10:3183–3197. 3183-3197. https://doi.org/10.18632/oncotarget.26900

