# Supplementary material for: Blood transcriptional profiling reveals IL-1 and integrin signaling pathways associated with clinical response to extracorporeal photopheresis in patients with leukemic cutaneous T-cell lymphoma
Source: Oncotarget. 2019 May 7;10(34):3183–97. doi: 10.18632/oncotarget.26900 (PMC6516711; doi:10.18632/oncotarget.26900)
Supplement: Supplementary file 1 [file oncotarget-10-3183-s001.pdf]

Blood transcriptional profiling reveals IL-1 and integrin signaling pathways associated with clinical response to extracorporeal photopheresis in patients with leukemic cutaneous T-cell lymphoma

SUPPLEMENTARY MATERIALS

Supplementary Table 1: List of RNA samples and microarray experiments

No.	Cy3	Cy5	Group	Donor	Clinical	BIS_name	Description
1	BL	D2	ND2	D1 (#1)	Non- responder	D1_N_D2vsBL	PBMCs at day2 post UV treatment relative to baseline
2	BL	M1	NM1			D1_N_M1vsBL	PBMCs at 1 month post UV treatment relative to baseline
3	BL	D2	ND2	C3 (#2)		C3_N_D2vsBL	PBMCs at day2 post UV treatment relative to baseline
4	BL	M1	NM1			C3_N_M1vsBL	PBMCs at 1 month post UV treatment relative to baseline
5	BL	D2	ND2	C4 (#3)		C4_N_D2vsBL	PBMCs at day2 post UV treatment relative to baseline
6	BL	M1	NM1			C4_N_M1vsBL	PBMCs at 1 month post UV treatment relative to baseline
7	BL	D2	ND2	C9 (#4)		C9_N_D2vsBL	PBMCs at day2 post UV treatment relative to baseline
8	BL	M1	NM1			C9_N_M1vsBL	PBMCs at 1 month post UV treatment relative to baseline
9	BL	D2	ND2	C13 (#5)		C13_N_ D2vsBL	PBMCs at day2 post UV treatment relative to baseline
10	BL	M1	NM1			C13_N_ M1vsBL	PBMCs at 1 month post UV treatment relative to baseline
11	BL	D2	RD2	C1 (#6)	Responder	C1_R_D2vsBL	PBMCs at day2 post UV treatment relative to baseline
12	BL	M1	RM1			C1_R_M1vsBL	PBMCs at 1 month post UV treatment relative to baseline
13	BL	D2	RD2	C2 (#7)		C2_R_D2vsBL	PBMCs at day2 post UV treatment relative to baseline
14	BL	M1	RM1			C2_R_M1vsBL	PBMCs at 1 month post UV treatment relative to baseline
15	BL	D2	RD2	C5 (#8)		C5_R_D2vsBL	PBMCs at day2 post UV treatment relative to baseline
16	BL	M1	RM1			C5_R_M1vsBL	PBMCs at 1 month post UV treatment relative to baseline
17	BL	D2	RD2	C14 (#9)		C14_R_ D2vsBL	PBMCs at day2 post UV treatment relative to baseline
18	BL	M1	RM1			C14_R_ M1vsBL	PBMCs at 1 month post UV treatment relative to baseline
19	BL	D2	RD2	C16 (#10)		C16_R_ D2vsBL	PBMCs at day2 post UV treatment relative to baseline
20	BL	M1	RM1			C16_R_ M1vsBL	PBMCs at 1 month post UV treatment relative to baseline

Abbreviations: BL: baseline; ND2: samples collected after treatment at Day 2 from patients resistant to ECP; NM1: samples collected after treatment at 1 month post-ECP from patients resistant to ECP; RD2: samples collected after treatment at Day 2 from patients responsive to ECP; RM1: samples collected after treatment at 1 month post-ECP from patients responsive to ECP.

Supplementary Table 2: Downregulated DEGs in RM1

Gene symbol	Systematic name	*p* value	Adj. *p* value	Fold changes	Log ratio
IL1B	NM_000576	7.85E-03	4.91E-01	-8.088	-3.016
EGR1	NM_001964	1.24E-02	4.99E-01	-7.624	-2.931
CCL3	NM_002983	9.29E-03	4.93E-01	-7.197	-2.847
CCL3L3	NM_001001437	1.34E-02	5.01E-01	-5.948	-2.572
CXCL2	NM_002089	8.66E-03	4.93E-01	-4.732	-2.243
CCL3L3	NM_001001437	2.86E-02	5.31E-01	-4.470	-2.160
GPR109B	NM_006018	4.32E-03	4.66E-01	-3.097	-1.631
IER3	NM_003897	2.59E-02	5.25E-01	-2.899	-1.536
THC2317149[*]	THC2317149	3.11E-02	5.42E-01	-2.600	-1.378
CD83	NM_004233	4.33E-03	4.66E-01	-2.531	-1.340
CXCL2	NM_002089	2.40E-02	5.24E-01	-2.358	-1.238
OTUD1	AB188491	9.55E-03	4.93E-01	-2.329	-1.220
ITGB3[*^]	NM_000212	3.77E-03	4.42E-01	-2.197	-1.136
LRRC2	NM_024512	4.74E-02	5.70E-01	-2.195	-1.134
TMEM107	NM_032354	4.14E-02	5.64E-01	-2.186	-1.128
PPP1R15A[*]	NM_014330	8.84E-03	4.93E-01	-2.185	-1.128
PTGS2	NM_000963	6.14E-03	4.89E-01	-2.034	-1.024
ENST00000238875	ENST00000238875	1.11E-02	4.99E-01	-1.978	-0.984
GNAZ[*]	NM_002073	1.51E-02	5.10E-01	-1.965	-0.975
GNG11[*]	NM_004126	2.99E-03	4.40E-01	-1.942	-0.957
PTGS2	NM_000963	9.97E-03	4.93E-01	-1.932	-0.950
GP1BA	J02940	4.50E-03	4.71E-01	-1.887	-0.916
CXCL1	NM_001511	4.32E-02	5.65E-01	-1.881	-0.912
ZFP36[*]	NM_003407	1.24E-02	4.99E-01	-1.876	-0.907
NRGN[*]	NM_006176	8.42E-03	4.92E-01	-1.853	-0.890
PPBP	NM_002704	3.26E-03	4.40E-01	-1.833	-0.874
SPARC	NM_003118	3.56E-02	5.47E-01	-1.824	-0.867
HSPC159	NM_014181	1.13E-02	4.99E-01	-1.809	-0.855
AXUD1	NM_033027	2.14E-02	5.24E-01	-1.808	-0.854
SDPR	NM_004657	1.40E-02	5.05E-01	-1.806	-0.853
C19orf33	NM_033520	2.31E-02	5.24E-01	-1.800	-0.848
CMTM5	NM_001037288	1.38E-02	5.03E-01	-1.793	-0.842
CLDN5	NM_003277	1.49E-02	5.09E-01	-1.787	-0.838
ITGA2B[*^]	NM_000419	4.93E-03	4.78E-01	-1.783	-0.835
THC2400010[*]	THC2400010	3.91E-03	4.45E-01	-1.777	-0.829
ITGB3[*^]	NM_000212	5.70E-03	4.89E-01	-1.774	-0.827
GP1BB	NM_000407	8.34E-03	4.91E-01	-1.767	-0.821
FLJ39779	NM_207442	2.94E-02	5.35E-01	-1.755	-0.812
SOD2	BC016934	3.05E-02	5.42E-01	-1.746	-0.804
TRIB1	NM_025195	2.34E-03	4.40E-01	-1.743	-0.802
AY358224	AY358224	4.88E-02	5.73E-01	-1.741	-0.800
LOC399900	NM_001013667	7.09E-03	4.90E-01	-1.741	-0.800
ENST00000299289[*^]	ENST00000299289	3.41E-02	5.47E-01	-1.726	-0.788
IER2	NM_004907	1.44E-02	5.06E-01	-1.720	-0.782
PDZK1IP1	NM_005764	3.90E-02	5.57E-01	-1.710	-0.774
ITGB5	NM_002213	3.64E-02	5.49E-01	-1.693	-0.760
ALOX12	NM_000697	2.32E-02	5.24E-01	-1.692	-0.759

*C5orf4**	NM_032385	2.48E-02	5.24E-01	-1.685	-0.752
THC2407148	THC2407148	2.61E-02	5.26E-01	-1.676	-0.745
GADD45B	NM_015675	4.58E-02	5.68E-01	-1.670	-0.740
MYL9	NM_181526	1.05E-02	4.97E-01	-1.667	-0.737
TUBB1	NM_030773	8.10E-03	4.91E-01	-1.657	-0.729
*RHOB**	NM_004040	4.94E-02	5.73E-01	-1.653	-0.725
*ITGA2B**^	NM_000419	6.06E-03	4.89E-01	-1.650	-0.723
SH3BGRL2	NM_031469	3.91E-02	5.57E-01	-1.650	-0.722
P2RY13	NM_023914	2.39E-02	5.24E-01	-1.649	-0.721
MOP-1	AB014771	4.38E-02	5.65E-01	-1.648	-0.720
TREML1	AY358357	7.23E-03	4.90E-01	-1.643	-0.716
BTG2	NM_006763	6.62E-03	4.89E-01	-1.641	-0.715
A_24_P315256	A_24_P315256	9.85E-03	4.93E-01	-1.638	-0.712
PMAIP1	NM_021127	4.32E-02	5.65E-01	-1.629	-0.704
TUBB1	NM_030773	5.16E-03	4.84E-01	-1.628	-0.703
*ENST00000299289**^	ENST00000299289	2.75E-02	5.30E-01	-1.623	-0.698
*HIST1H1C**	NM_005319	1.15E-02	4.99E-01	-1.598	-0.676
SLC24A3	NM_020689	2.08E-02	5.24E-01	-1.597	-0.675
*TUBA8**	NM_018943	1.24E-02	4.99E-01	-1.595	-0.673
BQ002790	BQ002790	4.09E-02	5.63E-01	-1.583	-0.663
FFAR2	NM_005306	2.73E-02	5.30E-01	-1.582	-0.661
HSPA1A	NM_005345	2.43E-02	5.24E-01	-1.573	-0.654
PTCRA	NM_138296	4.62E-02	5.68E-01	-1.572	-0.652
THC2377128	THC2377128	2.03E-02	5.24E-01	-1.566	-0.647
ABLIM3	NM_014945	9.01E-03	4.93E-01	-1.565	-0.647
AL522024	AL522024	1.02E-02	4.95E-01	-1.563	-0.644
ENST00000380946	ENST00000380946	3.82E-02	5.56E-01	-1.563	-0.644
*HIST1H3B**	NM_003537	1.95E-02	5.21E-01	-1.560	-0.642
PTGS1	NM_000962	2.42E-02	5.24E-01	-1.560	-0.642
A_32_P109645	A_32_P109645	3.04E-03	4.40E-01	-1.557	-0.639
CLU	NM_203339	1.30E-02	5.01E-01	-1.535	-0.618
MPL	NM_005373	2.91E-02	5.33E-01	-1.533	-0.617
ALOX12	NM_000697	3.13E-02	5.42E-01	-1.527	-0.610
PDZK1IP1	NM_005764	1.65E-02	5.15E-01	-1.526	-0.610
KLF6	NM_001300	4.86E-02	5.72E-01	-1.517	-0.601
PRKAR2B	NM_002736	3.61E-02	5.49E-01	-1.514	-0.598
VWF	NM_000552	1.68E-02	5.16E-01	-1.513	-0.597
F13A1	NM_000129	4.86E-03	4.78E-01	-1.513	-0.597
LTBP1	NM_206943	3.09E-02	5.42E-01	-1.512	-0.596
*CITED4**	NM_133467	2.41E-02	5.24E-01	-1.512	-0.596
LOC388114	BC036424	1.04E-03	4.40E-01	-1.511	-0.595
SNCA	NM_007308	4.90E-02	5.73E-01	-1.511	-0.595
AP1B1	NM_001127	1.11E-03	4.40E-01	-1.510	-0.595
CA431756	CA431756	4.55E-02	5.68E-01	-1.506	-0.591
ELOVL7	NM_024930	2.75E-02	5.30E-01	-1.504	-0.589
IGF2BP3	NM_006547	1.78E-02	5.19E-01	-1.501	-0.586
AB209345	AB209345	4.32E-02	5.65E-01	-1.501	-0.586

*DEG present in 2 groups. ^Two Agilent duplicate spots.

Supplementary Table 3: Upregulated DEGs in RM1

Gene symbol	Systematic name	*p* value	Adj. *p* value	Fold changes	Log ratio
AK023737	AK023737	3.20E-02	5.44E-01	1.689	0.756
USP34[*]	AL050376	3.92E-02	5.57E-01	1.682	0.751
AK024584	AK024584	4.53E-02	5.68E-01	1.610	0.687
POLR3E	AB040885	3.99E-02	5.61E-01	1.606	0.684
ZNF529	NM_020951	3.12E-03	4.40E-01	1.588	0.667
THC2431726	THC2431726	4.17E-02	5.64E-01	1.548	0.630
C22orf35	AK098753	3.37E-02	5.47E-01	1.546	0.629
BC029907	BC029907	3.54E-02	5.47E-01	1.546	0.629
CR620293	CR620293	2.28E-02	5.24E-01	1.538	0.621
C21orf66[*]	BC062992	3.78E-02	5.54E-01	1.529	0.613
BAT2D1	NM_015172	1.59E-02	5.13E-01	1.510	0.595

[*]DEG present in 2 groups.

Supplementary Table 4: Downregulated DEGs in RD2

Gene symbol	Systematic name	*p* value	Adj. *p* value	Fold changes	Log ratio
*THC2317149**	THC2317149	3.97E-02	3.71E-01	-2.315	-1.211
CD86	NM_006889	4.73E-02	3.82E-01	-2.102	-1.072
*GNAZ**	NM_002073	1.19E-02	3.01E-01	-2.089	-1.063
*ENST00000299289**^	ENST00000299289	1.63E-02	3.17E-01	-2.047	-1.033
HIST1H3D	NM_003530	2.23E-02	3.36E-01	-1.995	-0.996
*GNG11**	NM_004126	3.98E-02	3.71E-01	-1.924	-0.944
*ITGB3**^	NM_000212	2.81E-02	3.50E-01	-1.918	-0.939
*HIST1H3B**	NM_003537	9.78E-03	2.93E-01	-1.890	-0.918
THC2340803	THC2340803	4.02E-02	3.72E-01	-1.872	-0.905
*ENST00000299289**^	ENST00000299289	3.73E-02	3.67E-01	-1.818	-0.863
*THC2400010**	THC2400010	3.88E-02	3.69E-01	-1.788	-0.839
HIST1H3H	NM_003536	3.84E-02	3.69E-01	-1.775	-0.828
*ITGA2B**^	NM_000419	4.01E-02	3.72E-01	-1.741	-0.800
MAG	NM_080600	2.97E-02	3.53E-01	-1.671	-0.741
*NRGN**	NM_006176	3.48E-02	3.62E-01	-1.662	-0.733
*ITGA2B**^	NM_000419	1.22E-02	3.01E-01	-1.655	-0.726
*ITGB3**^	NM_000212	3.19E-02	3.57E-01	-1.638	-0.712
*TUBA8**	NM_018943	4.39E-02	3.79E-01	-1.638	-0.712
HIST1H2AE	NM_021052	1.18E-02	3.00E-01	-1.633	-0.707
HIST1H4H	NM_003543	1.15E-02	3.00E-01	-1.628	-0.703
IER5L	NM_203434	1.03E-02	2.94E-01	-1.607	-0.684
GMPR	NM_006877	2.87E-02	3.51E-01	-1.604	-0.682
*C5orf4**	NM_032385	4.30E-02	3.78E-01	-1.601	-0.679
*CITED4**	NM_133467	1.36E-02	3.10E-01	-1.578	-0.658
PPM1F	NM_014634	2.46E-02	3.43E-01	-1.575	-0.656
HIST1H2AM	NM_003514	7.63E-03	2.81E-01	-1.567	-0.648
HBG1	NM_000559	5.73E-03	2.75E-01	-1.558	-0.639
ANP32D	NM_012404	6.84E-03	2.78E-01	-1.557	-0.638
CHMP4B	NM_176812	1.01E-02	2.93E-01	-1.553	-0.635
*HIST1H1C**	NM_005319	3.36E-02	3.60E-01	-1.552	-0.635
RIN1	NM_004292	5.66E-03	2.75E-01	-1.535	-0.618
HIST1H2BH	NM_003524	2.23E-02	3.36E-01	-1.523	-0.607
HBG1	NM_000559	1.28E-02	3.05E-01	-1.522	-0.606
HIST1H2BO	NM_003527	9.00E-03	2.87E-01	-1.515	-0.599
LY6E	NM_002346	1.98E-02	3.27E-01	-1.512	-0.597
*RHOB**	NM_004040	3.76E-02	3.67E-01	-1.508	-0.593
C11orf17	NM_182901	3.90E-02	3.70E-01	-1.506	-0.590
METTL7A	NM_014033	2.27E-03	2.29E-01	-1.503	-0.588
UPK3A	NM_006953	4.17E-02	3.74E-01	-1.502	-0.587

*DEG present in 2 groups. ^Two Agilent duplicate spots.

Supplementary Table 5: Upregulated DEGs in RD2

Gene symbol	Systematic name	*p* value	Adj. *p* value	Fold changes	Log ratio
RGS1	NM_002922	1.65E-02	3.17E-01	2.343	1.228
BF089603	BF089603	2.34E-02	3.38E-01	2.269	1.182
ZNF331	NM_018555	1.98E-02	3.27E-01	1.907	0.931
*THC2400121**	THC2400121	2.44E-02	3.42E-01	1.823	0.866
PBEF1	NM_005746	4.56E-02	3.81E-01	1.792	0.842
*USP34**	AL050376	2.65E-02	3.47E-01	1.635	0.710
*C21orf66**	BC062992	9.71E-03	2.93E-01	1.627	0.702
*CD104030**	CD104030	4.37E-02	3.79E-01	1.616	0.693
ZNF331	NM_018555	3.78E-02	3.67E-01	1.614	0.691
EBI2	NM_004951	3.82E-02	3.69E-01	1.610	0.687
SCARNA17	NR_003003	1.12E-03	2.10E-01	1.570	0.651
ENST00000368491	ENST00000368491	6.44E-03	2.75E-01	1.560	0.642
LOC554208	AK098759	4.49E-02	3.80E-01	1.557	0.638
AK095242	AK095242	1.49E-02	3.11E-01	1.536	0.619
NAP1L5	NM_153757	1.87E-02	3.23E-01	1.533	0.616
LPIN1	AF147446	3.54E-03	2.50E-01	1.532	0.615
A_24_P899020	A_24_P899020	4.20E-02	3.75E-01	1.531	0.614
AK057576	AK057576	1.26E-03	2.10E-01	1.530	0.613
PNMA3	NM_013364	1.43E-02	3.11E-01	1.522	0.606
CXCR4	NM_001008540	2.12E-02	3.32E-01	1.518	0.602
DPY19L4	NM_181787	2.03E-04	2.10E-01	1.509	0.593
AKAP11	NM_144490	1.71E-02	3.19E-01	1.503	0.588
LOC153561	NM_207331	1.90E-02	3.23E-01	1.502	0.587

*DEG present in 2 groups.

Supplementary Table 6: Downregulated DEGs in NM1

Gene symbol	Systematic name	*p* value	Adj. *p* value	Fold changes	Log ratio
*THC2400121**	THC2400121	1.61E-02	5.15E-01	-1.917	-0.939
PHACTR1	AB051520	1.75E-02	5.15E-01	-1.762	-0.817
*ZFP36**	NM_003407	7.77E-03	5.07E-01	-1.686	-0.754
THC2271582	THC2271582	1.91E-02	5.18E-01	-1.637	-0.711
*PPP1R15A**	NM_014330	4.74E-02	5.68E-01	-1.604	-0.682
*CD104030**	CD104030	2.60E-02	5.37E-01	-1.600	-0.678
DUSP1	NM_004417	3.68E-02	5.54E-01	-1.582	-0.661
HBEGF	NM_001945	1.28E-02	5.15E-01	-1.564	-0.645
LOC349114	NM_198284	2.20E-02	5.30E-01	-1.550	-0.632
GABARAPL1	NM_031412	8.19E-03	5.07E-01	-1.532	-0.615
PLK3	NM_004073	1.92E-02	5.19E-01	-1.521	-0.605
EMD	NM_000117	3.71E-02	5.54E-01	-1.519	-0.603
THC2406011	THC2406011	1.36E-02	5.15E-01	-1.507	-0.591

*DEG present in 2 groups.

Supplementary Table 7: Upregulated DEGs in NM1

Gene symbol	Systematic name	*p* value	Adj. *p* value	Fold changes	Log ratio
BU561469	BU561469	4.55E-02	5.67E-01	1.607	0.684
A_23_P46070	A_23_P46070	1.23E-02	5.15E-01	1.575	0.655
RHO	NM_000539	9.81E-03	5.14E-01	1.533	0.616
HRASLS5	BC034222	1.15E-02	5.15E-01	1.529	0.612
THC2443137	THC2443137	1.46E-02	5.15E-01	1.517	0.601
A_32_P149404	A_32_P149404	3.85E-02	5.54E-01	1.505	0.590

Supplementary Table 8: Downregulated DEGs in ND2

Gene symbol	Systematic name	*p* value	Adj. *p* value	Fold changes	Log ratio
KSP37	NM_031950	1.33E-02	4.67E-01	-1.571	-0.652
AK098835	AK098835	2.49E-02	4.85E-01	-1.532	-0.615

Supplementary Table 9: Top DEGs in four groups analyzed

Group	RD2		RM1		ND2		NM1	
Top down-regulated DEGs	*CD86*	-2.102	*IL1B*	-8.088	*FGFBP2*	-1.571	*PHACTR1*	-1.762
	GNAZ[*]	-2.089	*EGR1*	-7.624	*ZEB2*	-1.532	*ZFP36*[*]	-1.686
	MPIG6B	-2.047	*CCL3*	-7.197	*MS4A4A*	-1.483	*SNORD3D*	-1.637
	HIST1H3D	-1.995	*CCL3L3*	-5.948	*FAM20A*	-1.463	*PPP1R15A*[*]	-1.604
	GNG11[*]	-1.924	*CXCL2*	-4.732	*FCGR3A/ FCGR3B*	-1.44	*DUSP1*	-1.582
	ITGB3[*]	-1.918	*HCAR3*	-3.097	*ADGRG1*	-1.429	*HBEGF*	-1.564
	HIST1H3B	-1.89	*IER3*	-2.899	*GZMB*	-1.428	*GABARAPL1*	-1.532
	HMBOX1	-1.872	*CD83*	-2.531	*SH2D1B*	-1.406	*PLK3*	-1.521
	HIST1H3H	-1.775	*OTUD1*	-2.329	*ERBB2*	-1.396	*EMD*	-1.519
	ITGA2B	-1.741	*ITGB3*[*]	-2.197	*PRSS23*	-1.395	*CTNNB1*	-1.484
	MAG	-1.671	*LRRC2*	-2.195	*FAM166B*	-1.387	*MEG3*	-1.452
	NRGN	-1.662	*TMEM107*	-2.186	*S1PR5*	-1.384	*GPR20*	-1.444
	TUBA8	-1.638	*PPP1R15A*[*]	-2.185	*TCF7L2*	-1.38	*RGCC*	-1.432
	HIST1H2AE	-1.633	*PTGS2*	-2.034	*AIF1*	-1.372	*IER2*	-1.421
	HIST1H4H	-1.628	*LGALSL*	-1.978	*B3GAT1*	-1.369	*SH2D1B*	-1.398
	IER5L	-1.607	*GNAZ*[*]	-1.965	*SLC1A7*	-1.365	*ZMYND15*	-1.398
	GMPR	-1.604	*GNG11*[*]	-1.942	*FNIP1*	-1.361	*SAT1*	-1.386
	FAXDC2	-1.601	*GP1BA*	-1.887	*GZMH*	-1.359	*ATG16L2*	-1.385
	CITED4	-1.578	*CXCL1*	-1.881	*GFOD1*	-1.352	*TSEN54*	-1.38
	PPM1F	-1.575	*ZFP36*[*]	-1.876	*SPON2*	-1.351	*PRPS1*	-1.376
Top up-regulated DEGs	*RGS1*	2.343	*AHSA2P*	1.682	*PLS3*	1.446	*RHO*	1.533
	IVNS1ABP	2.269	*POLR3E*	1.606	*TWIST1*	1.403	*HRASLS5*	1.529
	ZNF331	1.907	*ZNF529*	1.588	*OAZ1*	1.368	*ARHGEF15*	1.494
	NAMPT	1.792	*MIAT*	1.546	*KRT1*	1.367	*CCR3*	1.466
	AHSA2P	1.635	*PAXBP1*	1.529	*RGS13*	1.358	*PALM2-AKAP2*	1.453
	PAXBP1	1.627	*PRRC2C*	1.51	*RHO*	1.354	*CD19*	1.451
	GPR183	1.61	*CCDC14*	1.493	*HRASLS5*	1.339	*TMEM201*	1.419
	CEP85L	1.56	*HNRNPA3*	1.487	*ZNF557*	1.335	*BCL2*	1.395
	CTNNB1	1.536	*DPY19L4*	1.486	*PALM2-AKAP2*	1.329	*CSTF3*	1.391
	NAP1L5	1.533	*AHI1*	1.486	*TRIM35*	1.324	*NACC1*	1.385
	LPIN1	1.532	*LPIN1*	1.485	*FAM241A*	1.314	*TRIM49/ TRIM49C*	1.381
	PNMA3	1.522	*BICRA*	1.477	*TMEM201*	1.313	*SPATS2L*	1.373
	CXCR4	1.518	*PRPF4B*	1.477	*LLPH*	1.31	*ZNF595*	1.372
	DPY19L4	1.509	*PALM2-AKAP2*	1.474	*ARHGEF15*	1.307	*MACROD2*	1.371
	AKAP11	1.503	*PRC1*	1.466			*HERC6*	1.354
	ARL5B	1.499	*LLPH*	1.466			*DIS3*	1.326
	PHF20L1	1.497	*AKAP11*	1.458			*SLC6A19*	1.319
	CCDC14	1.495	*DIS3*	1.455			*HNRNPH2*	1.319
	GOLGA8A/ GOLGA8B[*]	1.494	*ANKRD23*	1.455			*BICRA*	1.318
	PAQR3	1.486	*GOLGA8A/ GOLGA8B*[*]	1.449			*ITM2A*	1.318

[*]DEG present in 2 groups.
